# Joint control of plant ecological strategy by climate, regeneration mode, and ontogeny in Northeastern Chinese forests

**DOI:** 10.1002/ece3.7522

**Published:** 2021-05-01

**Authors:** Xiangjun Zhang, Shuli Wang

**Affiliations:** ^1^ School of Forestry Northeast Forestry University Harbin China

**Keywords:** climate warming, CSR strategy, intraspecific variation, primary Korean pine forest, resprouting regeneration, seedling regeneration

## Abstract

1. Research on how plant ecological strategies (competitive, stress‐tolerant, or ruderal) vary within species may improve our understanding of plant and community responses to climate warming and also successional changes. With increasing temperature, the importance of ruderal (R) and stress tolerance (S) components is hypothesized to decrease, while the strength of the competitive (C) component should increase. Offshoots and younger plants are predicted to have greater R and smaller S components.
2. Leaf area, leaf dry matter content, and specific leaf area were measured for 1,344 forest plants belonging to 134 species in Liangshui and Fenglin Nature Reserves in Northeastern China, and C, R, and S scores calculated for each. Linear mixed effect models were used to assess how these indicators differed among study sites (*n* = 2), regeneration types, ontogenetic stages, and plant life forms. The two study sites have an average annual temperature difference of 0.675°C, simulating a temperature increase of 0.630°C due to climate warming.
3. Higher temperatures reduce low‐temperature stress and frost damage, which may explain the observed decrease in R and S scores; at the same time, plant competitive ability increased, as manifested by higher C scores. This effect was most pronounced for herbaceous plants, but nearly negligible as compared to the effect of regeneration type for trees and of ontogeny for woody species. Resprouting trees and younger woody plants had higher R scores and lower S scores, a sign of adaptation to high disturbance.
4. In this study, a small increase in mean annual temperature led to shifts in CSR strategy components for herbaceous species, without altering the vegetation type or community composition. Offshoots and younger plants had higher R and lower S scores, shedding light on similar changes in the ecological strategies of tree communities during secondary succession, such as the transition of *Quercus mongolica* coppices to forest and age‐related changes in *Populus davidiana*–*Betula platyphylla* forests.

## Graphical abstract

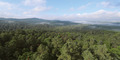

## 1 INTRODUCTION

Plant ecological strategy theory has been around for decades (Grime, 1974, 2001; Grime & Pierce, 2012; Pierce et al., 2017) and is widely applied to topics such as vegetation succession, the biodiversity–productivity relationship, plant distributions, ecological restoration, and species invasions (Aarssen, 2001; Dalle Fratte et al., 2019; Ikauniece et al., 2013; Jenkins & Pierce, 2017; Kienast et al., 1999; Rosenfield et al., 2019; Scolastri et al., 2017), although it has often been criticized (Tomáš et al., 2018; Wilson & Lee, 2000). The application of the theory has long suffered from the challenges associated with its quantitative implementation (Pierce et al., 2013, 2017). The early quantitative classification methods are complex and only applicable to native British herbs (Grime et al., 1997; Hodgson et al., 1999). More recently, functional traits have been used to classify plant species as competitive (C), stress‐tolerant (S), or ruderal (R), and this CSR classification method has been applied to over a thousand European species, including woody plants (Cerabolini et al., 2010; Mahmut et al., 2010; Pierce et al., 2017; Rosenfield et al., 2019). Pierce et al. (2017) created a globally calibrated CSR calculator using data on leaf area (LA), leaf dry matter content (LDMC), and specific leaf area (SLA) for 3,068 tracheophytes; this calculator renders CSR classification simpler and more effective. However, studies of CSR theory remain very limited in China (Wang et al., 2018), and the overwhelming majority of forest plant species in Northeast China are not represented in the aforementioned database of 3,068 species (fec12722‐sup‐0003‐tables1.xlsx) (Pierce et al., 2017); the CSR strategies of these species remain unknown.

Within‐species variability in CSR strategy also remains poorly characterized, yet may be significant. For example, species in Italy shows significant heterogeneity in CSR tertiary strategy (Astuti et al., 2019; Pierce et al., 2013). Climate‐induced shifts in plant species distributions and vegetation types may also affect ecological strategies within ecosystems (Gillison, 2019; Oliveira‐Filho et al., 2015; Pierce et al., 2017). For example, *Arabidopsis thaliana* shows a latitudinal gradient in ecological strategy as a result of within‐species adaptation to climate (Vasseur et al., 2018); stress‐tolerant strategies predominated at high latitudes in cold climates. Even along short temperature gradients, distinct forest types show clear differences in ecological strategy; at lower minimum temperatures, forests score more highly for stress tolerance, while warmer temperatures lead to greater competitiveness (Rosenfield et al., 2019). Similarly, alpine situations have experienced substantial periods of low temperature, in which competitors are lacking (Caccianiga et al., 2006), more species were stress tolerance (Cerabolini et al., 2016, Pierce et al., 2007). However, whether the slight changes in climate induced by short latitudinal gradients can cause variation in CSR strategy, without associated changes in vegetation type and community composition, remains unclear. In the context of global warming, studies on this topic will provide a better understanding of whether slight increases in temperature in localized areas can produce variability in plant adaptive strategies and if so, this variation should be quantified and compared to that caused by other factors, such as ontogenetic shifts.

Reports of intraspecific variability in plant CSR strategy caused by ontogenetic shifts are, however, very limited. In one study (Dayrell et al., 2018), juveniles exhibited higher SLA and LA and lower LDMC, indicative of a stronger R strategy to cope with high levels of disturbance. Greater variation in CSR strategy may be exhibited by juvenile plants, as they have not experienced ecological filtering in previous developmental stages, as is the case with adults. Previous studies have generally shown that leaf traits vary considerably during ontogeny (Niinemets, 2010; Steppe et al., 2011). The decrease in SLA shown during development is not as pronounced in pioneer tree species as compared to late successional species (Liang, 2017). A study of 38 tropical tree species (Rozendaal et al., 2006) found that short‐lived pioneers show the lowest plasticity in leaf traits as compared to shade‐tolerant species and long‐lived pioneers. In shade‐intolerant species (*Betula pendula* and *Populus tremula*), the leaf mass per unit area (LMA) increases more dramatically during ontogeny than it does for shade‐tolerant species (*Corylus avellana* and *Fagus sylvatica*) (Niinemets, 2006); the increase in LMA is mostly seen in the transition from seedling to sapling, with little further change occurring between saplings and canopy trees. These findings from different studies imply that intraspecific variability in tree CSR strategy, which also varies during development, may show complex changes among ontogenetic stages.

Resprouting plant species are typically associated with disturbance (Pausas & Keeley, 2014). When disturbance is frequent, trade‐offs in allocation to seedling versus resprouting regeneration are predicted to shift to the latter, with resprouting species being more common in repeatedly disturbed environments (Clarke & Dorji, 2008). Therefore, resprouting regeneration also plays an important role in forest restoration, particularly during the initial and mid‐successional stages; as succession proceeds, offshoots will gradually be replaced by seed‐established individuals (Kammesheidt, 1998; Simões & Marques, 2007). Resprouting species tend to have higher SLA and are more pioneer‐like than species that regenerate by seed alone (Anacker et al., 2011; Salk, 2012). According to CSR strategy theory, in comparison with undamaged plants of the same species, offshoots should be better adapted to disturbance, more pioneer‐like, and more common in early successional stages (i.e., should have a higher R score). However, CSR strategy theory has not been systematically applied to resprouting, and we still lack information on resprouting as a functional attribute (Bond & Midgley, 2003; Grime & Pierce, 2012). In this framework, comparison of C, S, and R components between seedlings and offshoots of the same species would improve our understanding of adaptive plant strategies and secondary succession.

Primary Korean pine forests occur naturally in the Xiaoxinganling and Changbai Mountains in Northeast China (Ma et al., 1992). Compared to forests of the southern subzone of Changbai Mountain and the northern subzone of the northern slope of Xiaoxingan Mountains, the primary Korean pine forest in the central subzone of the southern slope of Xiaoxingan Mountains is the most typical and representative of this type of forest, which is also known as typical Korean pine forest (Xu, 2001). In this study, natural Korean pine forests in the Fenglin Nature Reserve (located at the northern end of the southern slope of Xiaoxingan Mountains) and the Liangshui Nature Reserve (middle of the southern slope) were selected for study. These sites contained the most common type of primary Korean pine forest, mixed *Pinus koraiensis–Tilia amurensis* forests. For each forest site, the regeneration type (seedling or offshoot) and ontogenetic stage (juvenile, including seedlings and saplings, or adult, including young and mature individuals) were determined for samples of woody plant species, for which the CSR parameters were also calculated. This study addressed the following questions: (a) What are the CSR strategies of the main plant species in the primary Korean pine forest of Fenglin and Liangshui; (b) do plant CSR strategies vary between the two sites as a result of minor climatic differences; and (c) is there intraspecific variation in plant CSR strategy driven by ontogeny or regeneration type. We hypothesize that (a) variation in CSR strategy within plant species may be common and caused by even a minimal temperature difference; (b) offshoots and younger plants are predicted to show higher R and lower S components, a sign of better adaptation to disturbance.

## 2 MATERIALS AND METHODS

### 2.1 Study area

**FIGURE 1:**
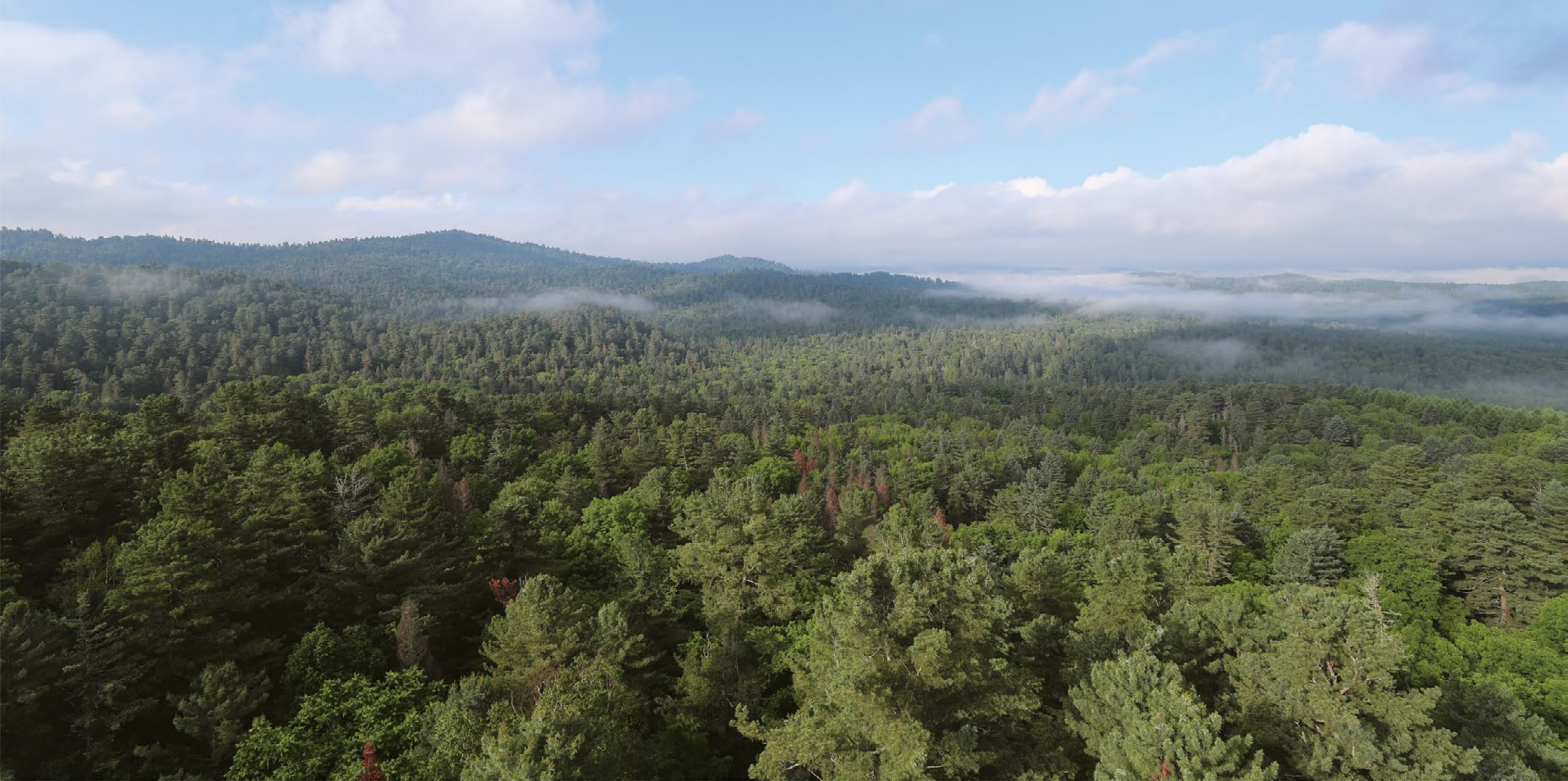
Primary Korean pine forest in Liangshui National Natural Reserve (taken by Dr Wei Gu, June 2020)

**FIGURE 2:**
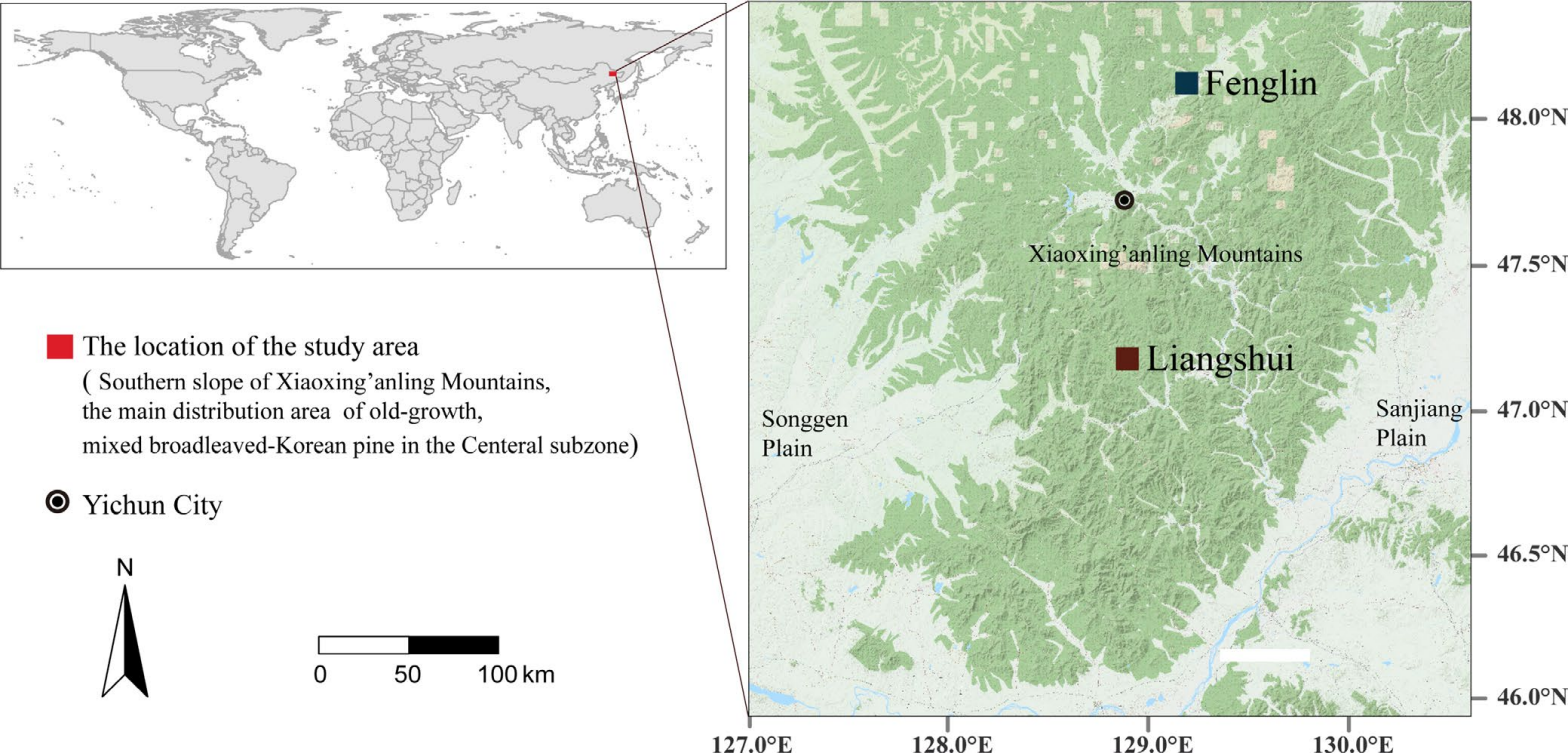
Location of the study areas in the Liangshui National Natural Reserve and Fenglin National Natural Reserve on the southern slope of the Xiaoxinganling Mountains, China, within the range of primary Korean pine forest in the central subzone

The research sites were situated within Liangshui National Natural Reserve (LS) (47°6′49″–47°16′10″N, 128°47′8″–128°57′19″E) and Fenglin National Natural Reserve (FL) (48°02′–48°12′N, 128°58′–129°15′E), both in the Xiaoxingan Mountains of Northeast China. The climate in this region is continental with monsoon features: In the spring, it is drier and windier. In both reserves, mixed *Pinus koraiensis* and *Tilia amurensis* forests are the predominant type of primary pine forest. These forests are diverse, containing hundreds of plant species. Forest soils are classified as dark brown forest soils, which are the typical soils took place under Korean pine forest. They are deep, fertile, and well‐drained. The research sites occurred at low altitudes (around 400 m above sea level) and mid‐slope on sunny slopes. By consulting forest archives and surveying, a *P. koraiensis*–*T. amurensis* forest was selected in both FL and LS for study (Figures 1 and 2). Plant leaf sampling occurred over a range of elevations that differed by no more than 20 m from that of the study *P. koraiensis*–*T. amurensis* forests; sometimes, this extra 20 m was required for adequate plant species sampling and replication, because resprouting individuals of *Alnus sibirica, Betula platyphylla*, and *Quercus mongolica* were challenging to sample. The mean annual precipitation (MAP) of the two sampling plots was essentially the same, while the mean annual temperature (MAT) differed slightly (Table 1). Precipitation and temperature data were downloaded from WorldClim (www.worldclim.org/bioclim).

**TABLE 1:** Basic characteristics of the study plots in Fenglin Nature Reserve and Liangshui Nature Reserve in Heilongjiang, China

Site	Geographical location	Altitude	Mean annual temperature	Mean annual precipitation
Fenglin	48°7′14″N 129°11′31″E	369 m	0.775°	626 mm
Liangshui	47°10′53″N 128°53′44″E	408 m	1.450°	627 mm

### 2.2 Experimental details

For both study forests, the leaves of all tree, shrub, and herbaceous species (a total of 134 species, including 25 trees, 28 shrubs, and 81 herbs, 123 species in FL and 120 in LS) were extensively sampled according to tree regeneration types and woody plant ontogenetic stages. For each type, the goal was to obtain at least six replicas per site; however, for a few rare plant species, only three to five replicates were possible. Very few plant species were too rare to be sampled sufficiently. In order to ensure enough regeneration samples (i.e., at least six replicates of each regeneration type per site) and to accurately identify resprouting versus seed regeneration, only five tree species were sampled (*A. sibirica*, *Acer mono*, *B. platyphylla*, *Padus racemose*, and *Q. mongolica*); additionally, sampled individuals including seedling and resprouting were mature trees that had flowered and fruited. For trees, four ontogenetic stages were established: seedling, sapling, young adult, and adult. Seedlings were defined for coniferous trees as individuals with height <30 cm and for broad‐leaved species as individuals with height <50 cm. Coniferous saplings had heights >30 cm and broad‐leaved sapling heights >50 cm, in addition to a diameter at breast height (DBH) < 5 cm. Young adult trees were those that had just entered the reproductive stage and generally had DBH ranging from 5 to 15 cm. Finally, adult trees were reproductively mature and had DBH > 30 cm. For shrubs, two ontogenetic stages were recognized: saplings and adults. Shrub saplings were structurally simple, unbranched individuals of height >50 cm that showed an absence of reproductive organs, while adults had entered the reproductive stage.

The leaf area (LA), leaf dry matter content (LDMC), and specific leaf area (SLA) were determined according to Pérez‐Harguindeguy et al. (2013). Leaf sampling took place from July 19 to 23, 2019, in LS, and from July 25 to 28, 2019, in FL; at this time, the leaves had fully expanded and reached maturity. Three to five unshaded directions were selected in the canopy for sampling, and vigorously growing branches were then collected for leaf extraction. Tall trees were sampled by professional climbers (Figure S1). Three to thirty undamaged, fully expanded leaves were measured per sampled individual. The C, S, and R scores for each individual were calculated using “StrateFy” (Pierce et al., 2017).

### 2.3 Data analysis

Four linear mixed effect models (LMMs) were constructed in order to assess variation in CSR scores and leaf trait values (i.e., log‐transformed LA, LDMC, and log‐transformed SLA) among plants with different life forms across sites, regeneration types, and ontogenetic stages. The first model had fixed effects of site (FL or LS) and life form (tree, shrub, or herb); in the second model, site and regeneration type (seedling or offshoot) were fixed effects. In the third model, site and ontogenetic stage (seedling, sapling, young adult, or adult) were fixed effects, while the fourth model had site, life form (tree or shrub), and ontogenetic stage (sapling or adult) as fixed effects.

As the influence of phylogeny could not be completely avoided, “Phylomatic” (http://phylodiversity.net/phylomatic/) was used to reconstruct species relationships (storedtree = Zanne et al., 2014) and to generate branch lengths for the phylogenetic tree (Webb & Donoghue, 2005; Zanne et al., 2014). In total, two phylogenetic trees were constructed: one for all 107 common plant species (across study sites FL and LS) and one for the 19 most common species at the sapling and adult stages. The phylogenetic signal in log‐transformed LA, LDMC, and log‐transformed SLA was examined separately for FL versus LS, and for saplings versus adults. Testing was performed with 100,000 randomizations using the multiPhylosignal function in the R package picante (Kembel et al., 2010).

The standard deviation (*SD*) of the C, S, and R scores was used as an indicator of variation in ecological strategy: a higher *SD* means a broader range of strategies (Dayrell et al., 2018). Wilcoxon rank sum tests were used to compare *SD*s between offshoots and seedlings, and saplings and adults; this approach also revealed the overall variation in CSR strategies. Furthermore, the sapling versus adult comparison was also made for shrubs and trees separately, to assess differences between these two life forms. Wilcoxon rank sum tests used the wilcox.test function in the R base package.

All statistical analyses were performed in R (version 4.0.2).

## 3 RESULTS

**FIGURE 3:**
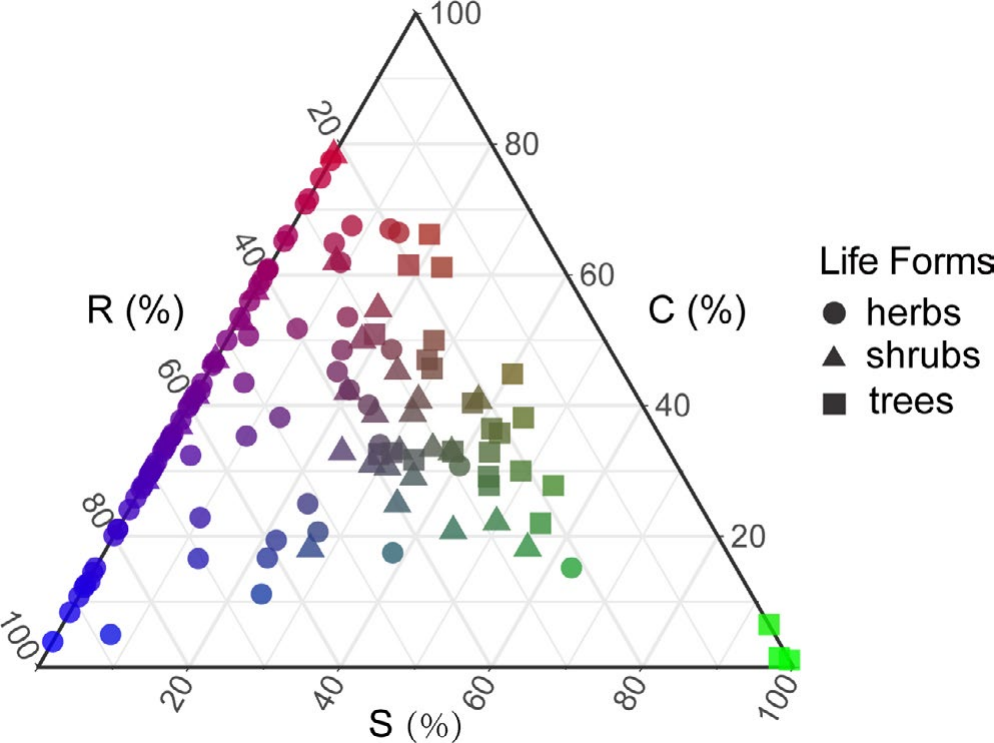
Ternary plot showing the relative proportions (%) of C, S, and R selection for 134 vascular plant species found in mixed Korean pine broad‐leaved natural forests in Northeastern China. Red indicates C‐selected, blue R‐selected, and green S‐selected species

**FIGURE 4:**
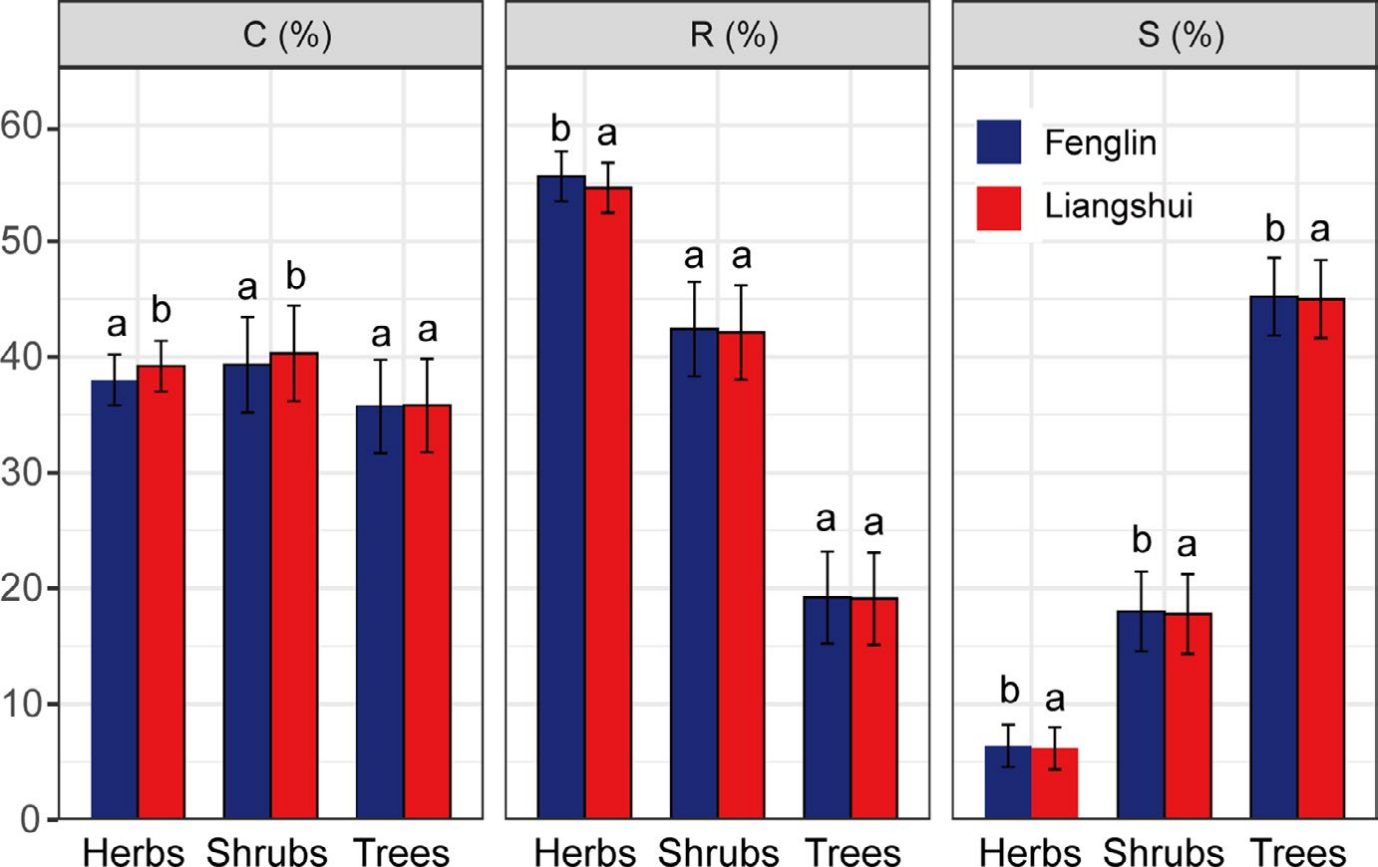
Variation in CSR scores between Fenglin (FL) and Liangshui (LS) study forests for three plant life forms (herbs, shrubs, and trees). Different letters indicate significant differences between FL and LS (*p* < .05). Bars represent estimated marginal means (sites | life forms)  ±  standard errors

A total of 134 species were positioned in a CSR strategy ternary diagram (Figure 3; Table S1). The C scores were higher in LS versus FL, while both R and S scores were lower. Comparing among life forms, the C, S, and R scores of herbaceous plants differed significantly between sites (*p* < .01, *p* < .05, and *p* < .01, respectively), while only the C (*p* < .01) and S (*p* < .05) scores of shrubs and the S score of trees (*p* < .05) differed significantly between sites (Figure 4; Tables S2 and S3). The log(LA) was higher in LS versus FL (*p* < .01), but log(SLA) and LDMC did not differ between sites (Tables S2 and S3). We found weakly significant phylogenetic signals for LA and LDMC in Liangshui (*K* = 0.384, *p = *.034; *K* = 0.876, *p = *.001) and Fenglin (*K* = 0.382, *p = *.029; *K* = 0.827, *p = *.001) (Table 2; Figure S2).

**TABLE 2:** Phylogenetic signal in leaf functional traits of the sampled Liangshui and Fenglin plants according to Bloomberg's *K*‐test

Classification	Leaf traits	*K*	*p*
Liangshui	LA	0.384	**.034**
	LDMC	0.876	**.001**
	SLA	0.242	.718
Fenglin	LA	0.382	**.029**
	LDMC	0.827	**.001**
	SLA	0.242	.697

Note

Significant *p*‐value (*p* < .05) marked in bold indicates that closely related species have trait values that are more similar than expected by chance.

**FIGURE 5:**
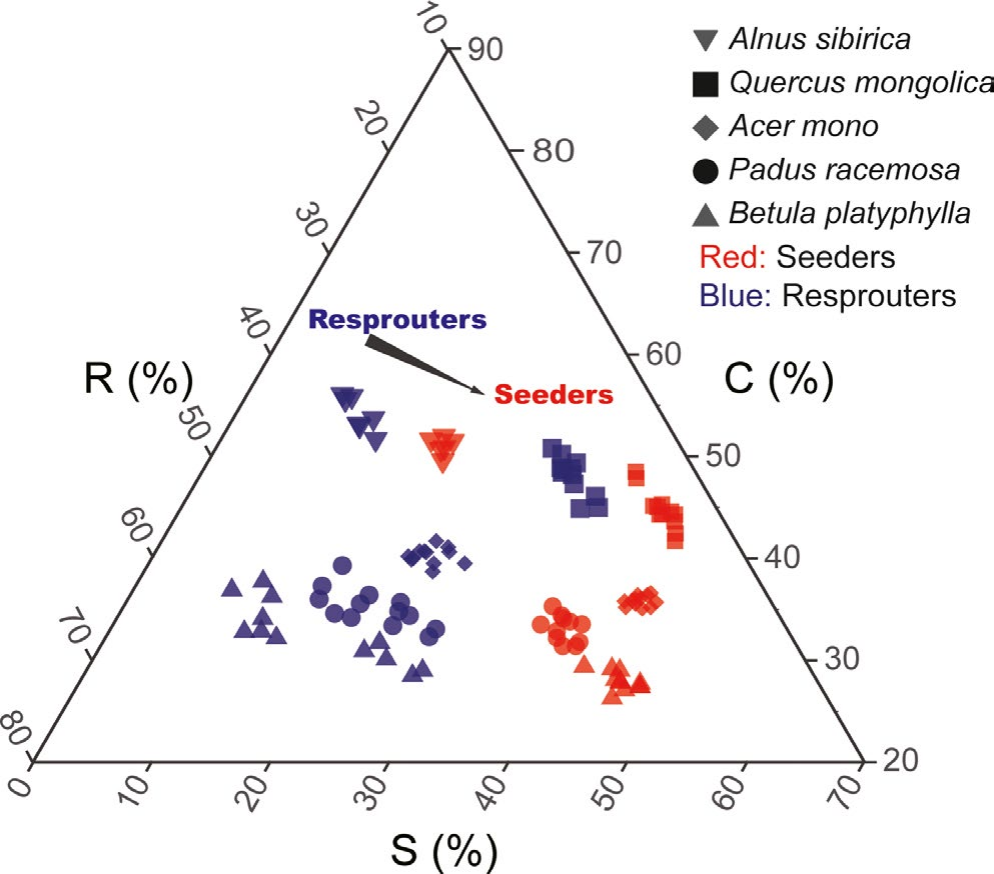
Ternary plot showing the relative proportions (%) of C, S, and R selection for seed‐derived versus resprouted individuals for five tree species

**FIGURE 6:**
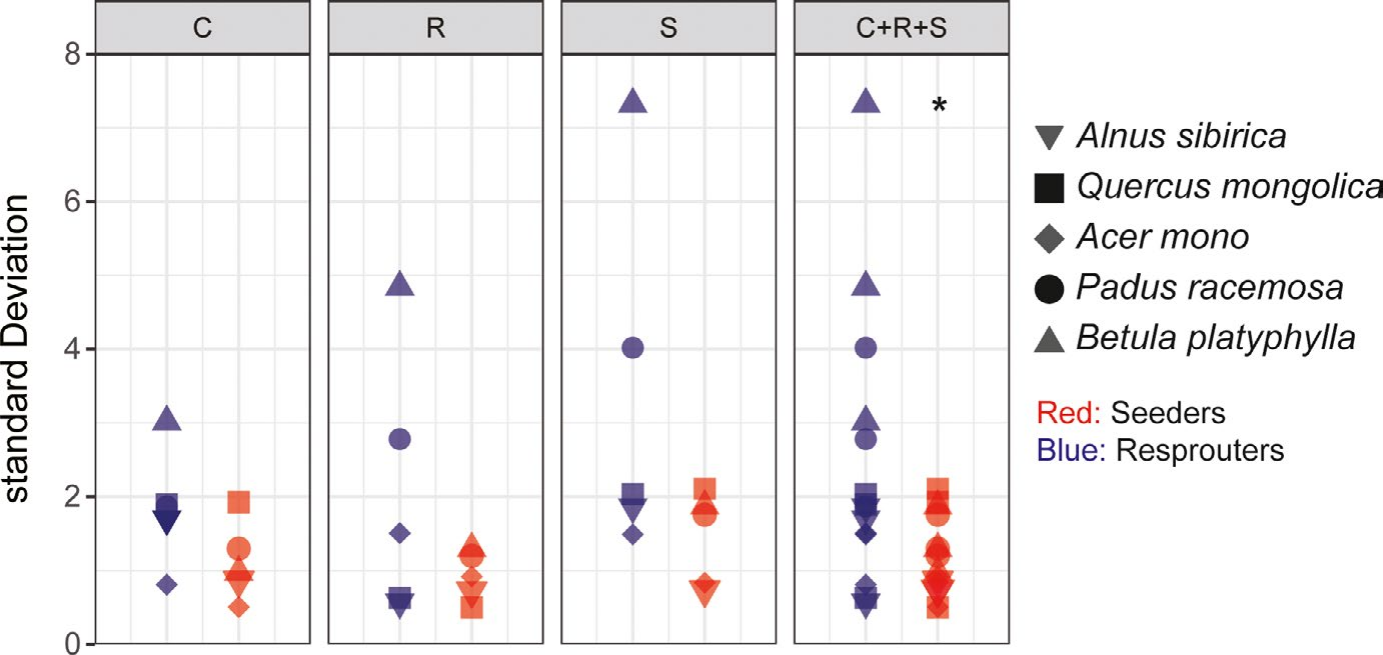
Standard deviation representing the variation in CSR scores between seed‐derived and resprouted individuals (seeders and resprouters, respectively). Asterisk indicates significant differences (*p < .05). A higher standard deviation represents a broader range of strategies

The C, S, and R scores, as well as the log(LA)** **(mm^2^), LDMC (%), and log(SLA) (mm^2^/mg), differed between new seedlings and offshoots (*p* < .01), but did not differ among sites; there were also no interactions between regeneration type and site. Offshoots had significantly higher C and R scores than seed‐established individuals (*p* < .01), but lower S scores (*p* < .01) (Figure 5; Tables S4 and S5). When considering all scores together (C + S + R), there was greater variation in strategy scores (i.e., higher SDs) in the resprouted vs. seed‐established individuals (*p* < .05) (Figure 6).

**FIGURE 7:**
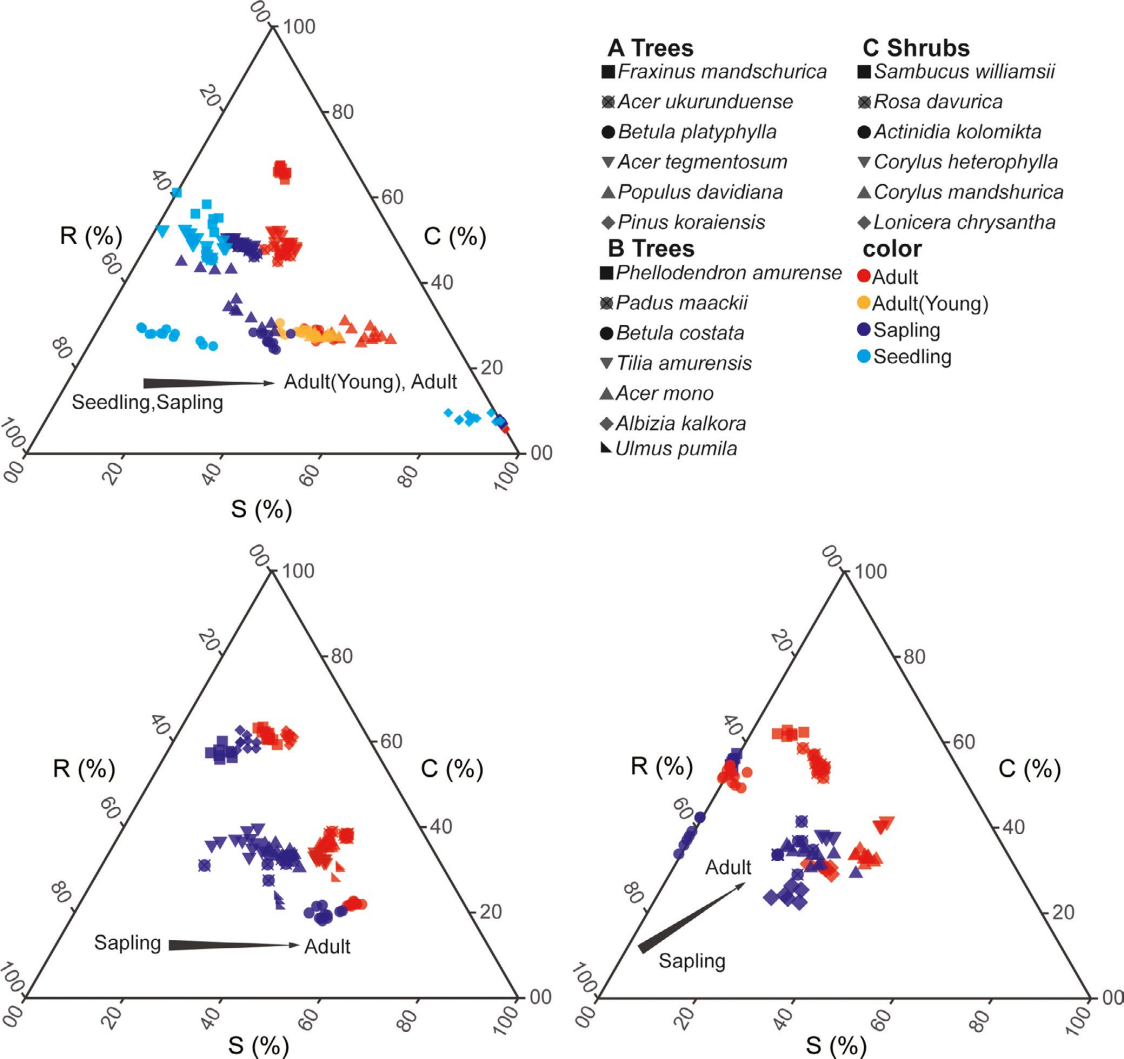
Ternary plot showing the relative proportions (%) of C, S, and R selection for different ontogenetic stages in tree and shrub species

The linear mixed effects model including ontogenetic stage (i.e., sapling, young adult, or adult) and site (i.e., FL or LS) as fixed effects found that only ontogeny had significant effects on C, S, and R scores, as well as the log(LA), LDMC, and log(SLA) (*p* < .01) (Figure 7a; Table S6). As plants matured, R scores continuously decreased (*p* < .01), while S scores increased (*p* < .05) (Figure 7a; Tables S6 and S7). The C scores of saplings were significantly higher than those of seedlings (*p* < .05), but there were no significant differences among the other stages (Figure 7a; Tables S6 and S7).

**FIGURE 8:**
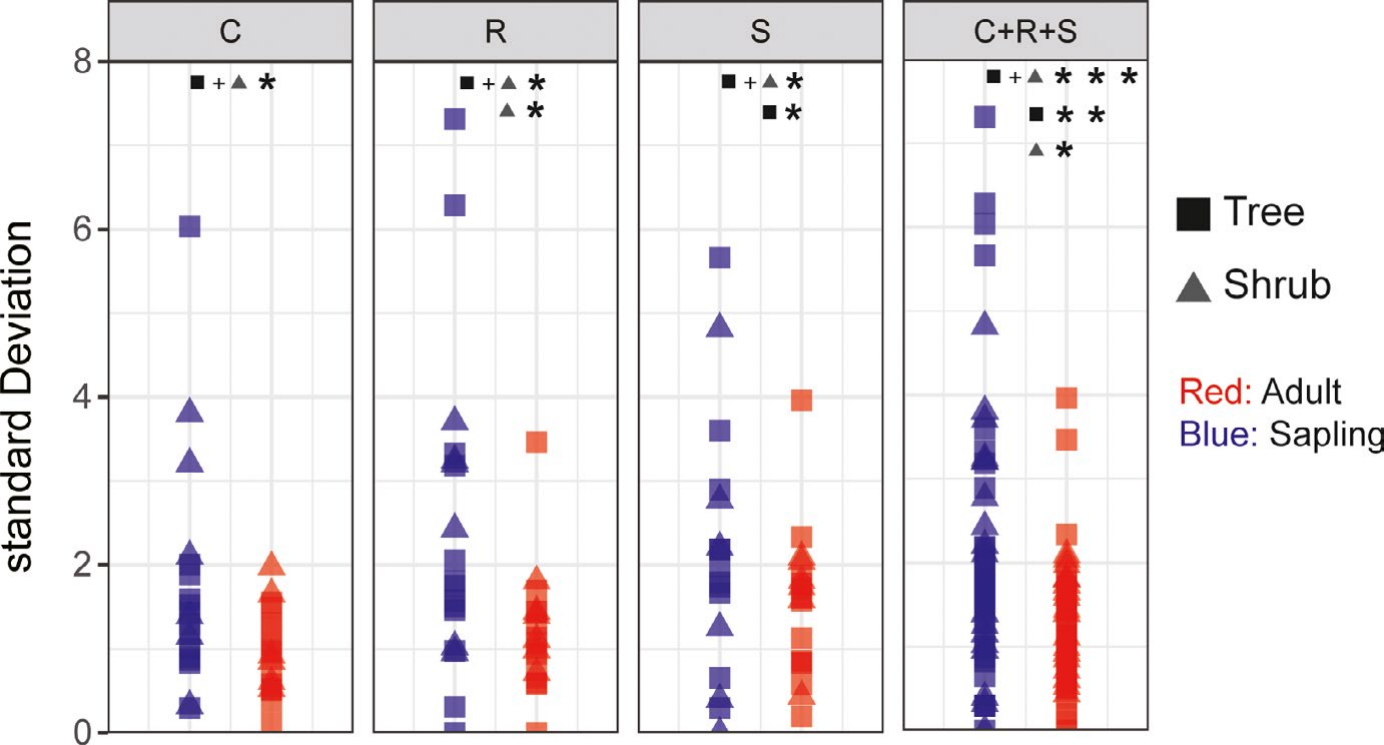
Standard deviation representing the variation in CSR scores between adult and sapling individuals. Asterisks indicate significant differences (*p < .05, **p < .01, and ***p < .001). A higher standard deviation represents a broader range of strategies

The linear mixed effects model including ontogenetic stage (i.e., sapling or adult), life form (i.e., tree or shrub), and site (i.e., FL or LS) as fixed effects revealed a significant interaction between ontogeny and life form (Figure 7b,c; Table S8). While R scores decreased with plant ontogenetic stage (*p* < .01), S scores increased (*p* < .01). Meanwhile, adult shrubs had higher C scores than shrub saplings (*p* < .01), but there was not an effect of ontogeny for the tree species (Figure 7; Tables S8 and S9). The C, S, and R scores (and combined C + S + R) were more variable in saplings versus adults (*p* < .05, *p* < .05, *p* < .05, and *p* < .001, respectively). Compared to adult individuals, R scores showed greater variation in shrub saplings (*p* < .05) and S scores were more variable in trees (*p* < .05) (Figure 8). Weak phylogenetic signals only were found for LDMC of adults (*K* = 0.501, *p = *.012) and juveniles (*K* = 0.461, *p = *.031) (Table 3; Figure S3).

**TABLE 3:** Phylogenetic signal in leaf functional traits of the sampled adult and sapling plants according to Bloomberg's *K*‐test

Classification	Leaf traits	*K*	*p*
Adults	LA	0.367	.052
	LDMC	0.501	**.012**
	SLA	0.184	.335
Saplings	LA	0.332	.114
	LDMC	0.461	**.031**
	SLA	0.402	.133

Note

Significant *p*‐value (*p* < .05) marked in bold indicates that closely related species have trait values that are more similar than expected by chance.

## 4 DISCUSSION

### 4.1 CSR strategies of primary Korean pine forest species

The simple and effective “StrateFy” tool created by Pierce et al. (2017) for calculating C, S, and R scores has been well tested and supported experimentally (Crowley & Wingler, 2020; Guo et al., 2019; Li & Shipley, 2017). However, application of this quantitative approach to natural systems has remained rather limited in China (Wang et al., 2018). To our knowledge, this is the first study to determine the CSR strategies of primary Korean pine forest species. In this study, 134 plant species were sampled and assessed; of the species considered, only *Convallaria majalis*, *Lonicera caerulea*, *Salix pentandra*, *Sorbaria sorbifolia*, *Spiraea salicifolia*, *Veratrum nigrum*, and *Viburnum opulus* were previously assessed by Pierce et al. (2017). The CSR strategy evaluations of *C. majalis* and *S. pentandra* do differ slightly here from the database values (*C. majalis* and *S. pentandra* are “CR” and “CSR,” respectively, in this study, and “CR/CSR” and “S/CSR” in the database). This discrepancy may be due to the small sample sizes for *C. majalis* (*n* = 6) and *S. pentandra* (*n* = 1), both rare species in this study. On the other hand, this finding may also support the hypothesis that within‐species variation in CSR strategy is common. In fact, Pierce et al. (2017) suggested that ecotypic variation across a species' range and phenotypic plasticity could broaden the range of strategic variation seen for a given species. Related studies have shown substantial variation along the S‐R axis for *Arabidopsis thaliana* populations occurring over a large geographical range (May et al., 2017; Vasseur et al., 2018). The endemic species *Bellevalia webbiana* also shows significant intraspecific variation in tertiary CSR strategy under disturbance (Astuti et al., 2019).

Among the 134 plant species considered in this study, CSR strategy varied along the S‐R axis, especially among life forms; coniferous trees had the lowest C and R scores, but the highest S scores. Compared to herbs and shrubs, canopy tree species had higher S scores, perhaps reflecting local sequestration of resources in long‐lived tissues in later successional stages (such as the *P. koraiensis–T. amurensis* forest); this phenomenon may lead to subsequent decreases in the amount of mineral nutrition available for plants (Grime, 2001). Additionally, low R selection is generally expected for woody species, as R‐selected species tend to have short life cycles and are mostly represented by small, fast‐growing annuals (Grime, 2001; Li & Shipley, 2017; Rosenfield et al., 2019). Some woody and herbaceous plant species in this study had relatively high C scores. This may be explained by occasional gaps in the canopy, improving light availability in local areas; the ensuing competition could favor C selection as succession proceeds (Pierce et al., 2017). Grime (2001) predicted that woody species varied from S (typically gymnosperm trees or angiosperm shrubs), through CS (angiosperm trees and woody lianas), to C (angiosperm trees with larger leaves) in the CSR triangle. Woody plants with CS or C strategies were not found in this study, perhaps due to the limited sampling within only one forest type; however, the general trend was similar (Figure 3). Previous studies have shown that herbaceous species fill the entire CSR ternary plot and may exhibit nineteen different CSR selection strategies (Cerabolini et al., 2010; Pierce et al., 2013). In the present study, the eighty species of herbs were found to belong to eleven CSR selection strategies, likely due to the smaller sample size.

### 4.2 Small increases in MAT produced shifts in CSR strategy

Few studies have focused on variation in leaf traits and CSR strategy across short latitudinal gradients, where vegetation type remains the same. However, this may be valuable information, especially in the context of global warming. Will regional warming lead to changes in plant adaptive strategies? The majority of previous studies involve larger geographical ranges or different vegetation types. Shifts from low to high latitude over long gradients produce major changes in environmental conditions (e.g., temperature and precipitation), affecting not only plant community richness and composition (Oliveira‐Filho et al., 2015), but also leaf traits (Gong et al., 2020; Luo et al., 2019; Zhang et al., 2019). As a result, the CSR strategies of individual plant species and the vegetation tend to shift dramatically (May et al., 2017; Pierce et al., 2017; Vasseur et al., 2018). Even along short latitudinal gradients, different forest types may show differences in ecological strategy (Rosenfield et al., 2019). In this study, the two research sites had a mean annual temperature difference of 0.675°C, with LS being warmer than FL. This difference in temperature is essentially the same as the climate warming seen in the region over the past 30 years, which saw an average increase of 0.021°C every year (You et al., 2015).

Despite the temperature difference, the MAP of the two study sites was essentially the same, though soil factors may have varied, given the large sampling area with varied site conditions. Thus, soil factors were not included among the independent variables considered in this study. As the MAT was higher (and the accumulated temperature greater) in LS, the growing season was also likely longer, potentially explaining the increase in LA at this site (Tables S2 and S3). Pierce et al. (2017) hypothesized that C‐selected species should have large individual and organ size (i.e., large LA) to aid in resource preemption, and Cerabolini et al. (2010) also associated C‐selected species with greater LA. In this study, only the C scores of herbs and shrubs differed significantly among sites; the invariance of tree species scores may have been due to the fact that the leaf traits of trees are also greatly affected by tree DBH and height (Martin & Thomas, 2013; Miyata & Kohyama, 2016). Herbaceous plants that are characterized by short‐lived, flimsy leaves are more strongly R‐selected than woody plant species and are also more sensitive to frost damage. Comparing study sites, FL is located at a higher latitude and has an earlier frost event, so plants especially for herbaceous plants there are more susceptible to frost damage (Zhang et al., 2014); this may explain why, in FL, only herbs had higher R scores (Pierce et al., 2013, 2017). This pattern is consistent with the decrease in S scores seen with large‐scale temperature increases; that is, temperature increases reduce pressure from low temperatures which directly limit plant growth rate by slowing metabolic processes in the growing season, and thereby decrease S scores (Vasseur et al., 2018).

### 4.3 Variation in CSR strategy driven by regeneration type

Regeneration via resprouting has not been included in traditional CSR strategy theory (Bond & Midgley, 2003; Grime & Pierce, 2012), nor has it received much research attention. Yet within‐species studies of how CSR strategy responds to regeneration type (whether vegetative or by seed) could improve our understanding of plant adaptation to disturbance and its role in vegetation restoration and succession (Bond & Midgley, 2001; Pausas & Keeley, 2014). For example, one type of primary Korean pine forest, mixed *Q. mongolica–P. koraiensis* forests, degenerates into pure *Q. mongolica* forests when disturbed (e.g., by fire); repeated fire events lead to the degradation of the forest into coppices (Zhou, 1994). As inferred from CSR strategy theory, resprouting *Q. mongolica* trees and stands, which are disturbance‐adapted and appear at early successional stages, should have higher R and lower S scores (Grime & Pierce, 2012; Pierce et al., 2017). This hypothesis received support here for several species, including *A. mono*, *A. sibirica*, *B. platyphylla*, *P. racemose*, and *Q. mongolica* (Figure 5; Tables S4 and S5). Offshoots not only had higher R and lower S scores, but also had also higher C scores (Figure 5; Tables S4 and S5).

The variation in CSR strategy seen for resprouted individuals is likely due to larger LA, smaller LDMC, and higher SLA (Tables S4 and S5). Resprouted trees already have an established root system, an advantage in acquiring water and other resources, allowing them to grow faster and have a competitive advantage (Dietze & Clark, 2008; Mostacedo et al., 2009; Simões & Marques, 2007). In other words, resprouted trees are under stronger C selection than conspecific nonresprouted individuals. Previous studies have shown that resprouted trees have greater LAs and SLAs than nonresprouted conspecifics; they act more like R‐selected, pioneer species (Kauppi et al., 1990; Salk, 2012). Across plant species, resprouted individuals also show higher SLAs than nonresprouted individuals (Ackerly, 2003; Paula & Pausas, 2006). These findings are basically consistent with those studies. However, the effects of regeneration type on leaf traits can be remarkably complicated. In a coastal shrubland, the LDMC of 30 woody species was significantly higher in resprouted individuals than in seedlings (Saura‐Mas & Lloret, 2007). Other studies have found few consistent differences in LA between species that can regenerate via resprouting and those that do so only via seed (Knox & Clarke, 2005). Owing to sampling challenges in this study, only five tree species capable of both vegetative and seed regeneration were evaluated. Most offshoots were located at forest edges or in gaps, under conditions of sufficient light but high disturbance; offshoots occurred during the early stages of vegetation restoration in micro‐environments. Meanwhile, conspecific, adult seed‐derived trees have experienced the process of succession and continuous trait filtering throughout the course of development (Dayrell et al., 2018). As a result, seed‐derived trees exhibited stronger conservation, higher S scores, and smaller intraspecific variability in strategy than resprouted trees did (Figure 6). The height of resprouted trees did not exceed five meters and most had the shape of tufted shrubs; these characteristics were important for discriminating regeneration type. Originally, the study plan included shrubs, but this was abandoned because it was not possible to accurately distinguish between resprouted and seed‐derived shrubs. In the future, the role of regeneration type in driving CSR strategy should be investigated in more woody plant species (both trees and shrubs), to better characterize the adaptive mechanisms used by plants of different life forms and their role in ecological succession.

### 4.4 Variation in CSR strategy during ontogeny

Due to environmental filters and selection pressures, juveniles tend to stronger R components than conspecific adults (Dayrell et al., 2018). Apart from this finding, there has been no further work on strategy variation during ontogeny. Leaf traits of tree species with different ecological strategies (succession stage, shade tolerance) show complex changes throughout plant development and at different stages of maturity (Lasky et al., 2015; Lusk & Warton, 2007; Niinemets, 2006, 2010; Pu et al., 2020; Rozendaal et al., 2006). Therefore, greater complexity in intraspecific variation for CSR strategy might be expected in a complete plant life cycle, from seedling, to sapling, to mature individual. For example, mixed poplar‐birch forests occur early in the secondary succession of primary Korean pine forests. According to CSR strategy theory, poplar‐birch forests and their dominant tree species (*Populus davidiana* and *Betula platyphylla*) should therefore experience stronger R selection than S selection when young (Grime, 2001; Grime & Pierce, 2012; Pierce et al., 2017). As trees mature, this pattern reverses. In this study, R scores continuously decreased (*p* < .01), while S scores increased (*p* < .05), as plants matured from seedlings to saplings, to mature plants (Figure 8a; Tables S6 and S7), in support of the ontogeny‐related hypotheses of CSR strategy theory.

Overall, C scores largely remained unchanged during ontogeny in trees, but did increase with ontogenetic stage in shrubs (Figure 7; Tables S6‐S9). However, there was great variation among species underlying these general trends. The pinnately compound leaves of mature *Phellodendron amurense* and *Fraxinus mandshurica* are larger compared to those of juveniles, and the C scores for these species also increased with ontogenetic stage; however, the opposite pattern was observed for *P. davidiana* and *P. koraiensis* (Figure 7a,b). Among the shrub species, the C score of *Corylus mandshurica* was virtually unaffected by ontogeny (Figure 7c). Previous work has shown that C‐selected species show wider ranges of trait intraspecific variation and exhibit higher phenotypic plasticity (Albert et al., 2011; Astuti et al., 2019). Comparing trees and shrubs, the difference in C score response may be that shrubs more occurred in productive microhabitats and trees in unproductive ones for our study. In productive habitats, ecological strategy may tend to shift toward C selection over the lifespan, while it may shift toward S selection in unproductive habitats (Dayrell et al., 2018). In this study, the adult individuals of *P. amurense* and *F. mandshurica* had the largest LAs (on average 20,747.3 mm^2^ and 30,948.2 mm^2^, respectively) and the highest C scores (on average 61.5 and 66.2, respectively). However, juveniles of these species may have been too small to have sufficiently large LA for a competitive strategy; this might be the same for most of the shrub species. Plants are exposed to multiple environmental stresses throughout their lifetime, the effects of which may be influenced by factors such as past stress history, stress interactions, tolerance, and acclimation (Niinemets, 2010; Valladares & Pearcy, 2002). More research needs to be done to better understand variations in C scores during ontogeny.

Previous studies have shown phylogenetic nonindependence in leaf traits (Mason et al., 2016). Although weakly significant phylogenetic signals were found for LA and LDMC in this study (Tables 2 and 3), phylogenetic effects are unlikely to have played a significant role (Dayrell et al., 2018). Instead, the slight difference in MAT between LS and FL most likely underlay the observed CSR strategy variation, especially for herbaceous plants. However, for woody plants, no significant strategy differences were found between FL and LS, at least as compared to the variation caused by regeneration type and ontogeny; thus, this variation can safely be ignored.

## 5 CONCLUSIONS

This study assessed for the first time the CSR strategies of primary Korean pine forest species and quantitatively evaluated strategy variation caused by differences in MAT, regeneration type and ontogenetic stage. Overall, variation in CSR strategy was common within plant species. In LS versus FL, higher temperatures produced lower R and S scores and increased plant competitive ability (C scores), especially for herbs. But the effect of temperature was negligible compared to the effects of regeneration type in trees and ontogenetic shifts in woody species. Resprouting trees and younger woody plants had higher R and lower S scores, probably as adaptation to disturbance. This study should contribute to our understanding of plant adaptive strategy shifts caused by regional warming and of ecological strategy shifts in tree communities (e.g., Mongolian oak, and poplar‐birch forests) during secondary succession.

## CONFLICT OF INTEREST

The authors declare no conflict of interest.

## AUTHOR CONTRIBUTIONS

**Xiangjun Zhang:** Conceptualization (lead); data curation (lead); formal analysis (lead); funding acquisition (supporting); investigation (lead); methodology (equal); project administration (supporting); resources (supporting); software (supporting); supervision (supporting); validation (supporting); visualization (equal); writing‐original draft (lead); writing‐review & editing (equal). **Shuli Wang:** Conceptualization (supporting); data curation (supporting); formal analysis (supporting); funding acquisition (lead); investigation (supporting); methodology (equal); project administration (lead); resources (lead); software (lead); supervision (lead); validation (lead); visualization (equal); writing‐original draft (supporting); writing‐review & editing (equal).

### OPEN RESEARCH BADGES

This article has earned an Open Data Badge for making publicly available the digitally‐shareable data necessary to reproduce the reported results. The data is available at https://doi.org/10.5061/dryad.bvq83bk8f.

## Supporting information

Supplementary Material

## Data Availability

Data are available at https://datadryad.org/stash/share/b5eMdXhmPKs3blIjOSL1PfPoeyI5K_f629YF4go4uuw (https://doi.org/10.5061/dryad.bvq83bk8f).
